# Targeted delivery of extracellular vesicles in heart injury

**DOI:** 10.7150/thno.51571

**Published:** 2021-01-01

**Authors:** Peier Chen, Ling Wang, Xianglin Fan, Xiaodong Ning, Bin Yu, Caiwen Ou, Minsheng Chen

**Affiliations:** 1 Laboratory of Heart Center and Department of Cardiology, Zhujiang Hospital, Southern Medical University, Guangzhou, China.; 2 Guangdong Provincial Biomedical Engineering Technology Research Center for Cardiovascular Diseases, Guangzhou, China.; 3 Laboratory of Heart Center, Sino-Japanese Cooperation Platform for Translational Research in Heart Failure, Guangzhou, China.; 4 Biomaterials Research Center, School of Biomedical Engineering, Southern Medical University, Guangzhou 510515, China.

**Keywords:** Extracellular Vesicles, Biogenesis, Heart Injury, Challenges, Targeted Delivery

## Abstract

Extracellular vesicles (EVs) are nanoscale extracellular vesicles derived from endocytosis that are crucial to intercellular communication. EVs possess natural biocompatibility and stability that allow them to cross biological membranes and that protect them from degradation. Recent studies have shown that EVs-mediated crosstalk between different cell types in the heart could play important roles in the maintenance of cardiac homeostasis and the pathogenesis of heart diseases. In particular, EVs secreted by different types of stem cells exhibit cardioprotective effects. However, numerous studies have shown that intravenously injected EVs are quickly cleared by macrophages of the mononuclear phagocyte system (MPS) and preferentially accumulate in MPS organs such as the liver, spleen, and lung. In this review, we discuss exosome biogenesis, the role of EVs in heart diseases, and challenges in delivering EVs to the heart. Furthermore, we extensively discuss the targeted delivery of EVs for treating ischemic heart disease. These understandings will aid in the development of effective treatment strategies for heart diseases.

## Introduction

Heart diseases remain the leading causes of death or disability in the world 1, 2. Stem cell transplantation may be an effective way to improve and treat acute and chronic ischemic heart disease 3, 4. However, the therapeutic potential of transplanted stem cells is limited by their low survival rate following transplantation into damaged myocardium. Also, arrhythmias and myocardial hypertrophy are prone to occur after stem cell transplantation, and there is a risk of cancer development 5, 6.

Many studies have shown that stem cell-derived extracellular vesicles (EVs) have the same myocardial repair function as transplanted stem cells 7-9. EVs are nanoscale vesicles secreted by almost all cells in the body with a lipid double-layer membrane 10. EVs are widely distributed in blood, cerebrospinal fluid, saliva, amniotic fluid, urine, and other body fluids 11, 12. In 1987, Johnstone and colleagues 13, 14 identified the maturation process of red blood cells (RBCs) in sheep. They showed that the transferrin receptor located on the immature RBC membrane is transferred from the cell membrane to the membrane of EVs secreted by erythrocytes during RBC maturation. In recent years, studies have shown that EVs carry some essential signaling molecules, such as DNAs, proteins, lipids, mRNAs, miRNAs, and siRNAs 15, and that they can mediate signal transmission between cells 16-18. Moreover, EVs play an important role in cardiovascular pathophysiology 19, 20. EVs have the following advantages over stem cells in the treatment of heart diseases: 1) EVs lack self-replicating entities; therefore, they have no tumorigenic potential 21-23; 2) EVs preserve their contents and their functions are relatively stable 24; 3) EVs can cross biological barriers, so it is easier for them to reach the area of ischemic injury 25; 4) EVs can be easily modified and stored 26; 5) EVs have the biological characteristics of their source cells and can carry a variety of bioactive molecules to act on receptor cells 27; and, 6) EVs can help cells clear misfolded prion proteins 28. EVs also play essential roles in the interactions between different cell types in the microenvironment of heart diseases. Bang *et al*. 29 investigated the potential paracrine miRNA crosstalk between cardiac fibroblasts and cardiomyocytes and found that cardiac fibroblasts secrete miRNA-enriched EVs. Through confocal imaging and co-culture experiments, they determined that miR-21 derived from fibroblast EVs is an effective paracrine RNA molecule that can induce cardiomyocyte hypertrophy. Following cardiac injury, EVs are rapidly released in a remarkable quantity to the local microenvironment. Cheng *et al*. 30 found that EVs secreted by ischemic cardiomyocytes are enriched in miR-1 and miR-133, which affect action potentials and cardiac conduction through targeting of Ca^2+^/calmodulin-dependent protein kinase II 31. In the cardiomyopathy associated with type 2 diabetes, Wang *et al.* 32 indicated that cardiomyocytes exert an anti-angiogenic function in type 2 diabetic rats through EVs-mediated transfer of miR-320 into endothelial cells. Non-stem cells-derived EVs can also mediate intercellular communication and provide cardioprotective effects. For example, under glucose deprivation conditions, EVs derived from cardiomyocytes were found to promote angiogenesis of endothelial cells 33.

EVs have a tremendous potential to replace stem cells and tissue-engineered therapies. However, the main limitation of this application is engraftment of EVs at the target site. Delivering a therapeutic dosage of EVs to the target site, particularly via systemic injection, can be challenging. Therefore, it is of paramount importance to explore strategies for targeted therapeutic delivery of EVs to the heart in order to devise more unique and valid treatment strategies for heart diseases.

## Biogenesis of Extracellular Vesicles

**Figure 1:**
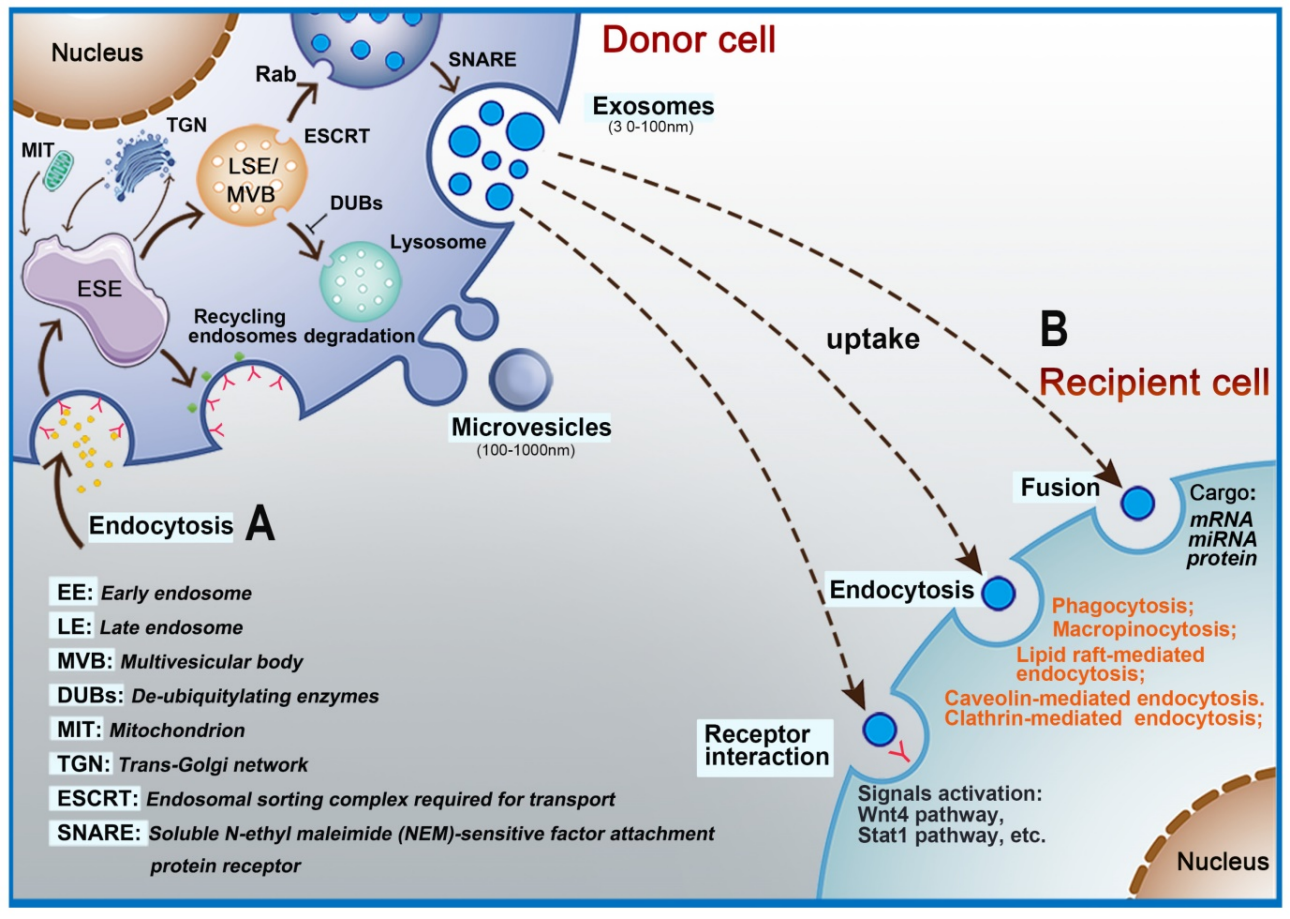
** Intracellular biogenesis and secretion of extracellular vesicles.** A: The process of exosome formation begins with early-sorting endosomes (ESE) formed by endocytosis on the surface of plasma membranes. Subsequently, ESE matures to generate a late endosome (LSE)/multivesicular body (MVB) and exosomes are released into the extracellular space by fusion of MVBs. B: Exosomes interact with target cells via receptors, fusion with plasma membrane, endocytosis, or release of their cargo.

EVs are nanoscale bubble-like membranous structures secreted by cells 34, 35. Exosomes, with an average diameter of 30-120 nm, are a subset of EVs 36. Exosomes carry a variety of biologically active substances (e.g., proteins, lipids, nucleic acids) that can affect the biological function of recipient cells and so exosomes are a way for cells to interact. Exosome formation is a complex process 37. It begins with the formation of early-sorting endosomes (ESE), which are created by fusion of primary endocytic vesicles 38-40. The trans-Golgi endoplasmic reticulum promotes the formation of ESE 41-43. ESE can return to the plasma membrane as the “recycling endosome” or mature to generate a late endosome (LSE)/multivesicular body (MVB) 18, 39. The formation of MVB requires assistance from the endosomal sorting complex required for transport (ESCRT). ESCRT is a protein complex located on the cytoplasmic side of the endosome and its main role is to sort specific components into ILVs, which in turn constitute the precursors of exosomes 44. The ESCRT apparatus comprises four types of complexes (ESCRT- 0, I, II, and III) and accessory proteins (VPS4, VTA1, ALIX, etc.), each playing diverse regulatory roles. ESCRT-0 is responsible for the recognition and separation of ubiquitin-labeled endosomal transmembrane proteins 45-47. ESCRT-I can be connected to ESCRT-II, and the two entities germinate inward to promote absorption of corresponding secretions by the endocytic membrane 48. Subsequently, ESCRT-III binds to the corresponding complex and communicates with the cell membrane to release buds, which then enter endosomes in the cell 49. If the cargo are deubiquitinated by deubiquitinating enzymes (DUBs), the goal is delivery to lysosomes for degradation 50. The Rab family are small GTPase proteins that control the transport processes of intracellular vesicles, such as movement of vesicles through the cytoskeleton and positioning of vesicles on the plasma membrane 51. Studies have shown that Rab11, Rab35, Rab27A/B, and Rab9 are related to the secretion of exosomes 48, 52-54. Soluble *N*-ethyl maleimide (NEM)-sensitive factor attachment protein receptor (SNARE) is a protein complex that can fuse plasma membranes that are in contact with each other and also promote the fusion of vesicle membranes and cell plasma membranes 55, 56. Cells can also generate LVs and MVBs without relying on the ESCRT pathway, and assist in the generation of lipids, ceramides, tetraspanins, and heat shock proteins 57 **(Figure 1A)**. Microvesicles are released into the extracellular space through outward sprouting and fission of plasma membrane 58.

EVs contain proteins, lipids, and nucleic acids 40 that are closely related to the donor cell and powerfully influence the recipient cell 59, 60. Depending on the origin of exosomes, protein components include typical transmembrane proteins that cross the membrane such as LAMP1/2, CD13, PGRL, and trafficking membrane proteins such as annexin and RABs. The lipid bilayer membrane of EVs also has a complex structure and mainly contains adhesion molecules such as ICAM-1 and other proteins such as LBPA, flotillins, cholesterol, tetraspanins, and stomatin that can affect lipid rafts 61. CD9, CD63, CD81, and CD82, which are present on the membrane, are commonly used as markers of exosomes. Additionally, the EVs membrane surface is assembled with immunomodulatory molecules such as MHC-I/II 62, 63. In the exosome cavity, several proteins have been found that stabilize and preserve the “informative” exosome cargo: HSP, cytoskeletal protein, metabolic enzymes (ATPase, GAPDH, elongation factors, and pgk1), and cytoskeletal proteins (tublin, actin, vimentin, cofilin, moesin, and talin) 64, 65 **(Figure 3A)**.

The contents of EVs that can be transferred from donor cells to target cells in the microenvironment include RNAs and a large number of proteins depending on the recipient cells 40, 66, 67. EVs have also been shown to contribute to intercellular communication by passing signal molecules or by surface-expressed ligands. Interactions between EVs and recipient cells are highly intriguing. Depending on their contents and the type of recipient cell, EVs transfer their cargo to recipient cells by fusing with the plasma membrane through receptors or by endocytosis** (Figure 1B)** 68, 69. For example, EVs (especially exosomes) promote communication between neural stem/precursor cells and the microenvironment through receptor-ligand interactions 70. Many key proteins that promote EVs uptake have been discovered, including the tetraspanin membrane proteins CD9 and CD81 and intercellular adhesion molecule (ICAM)-1 71, 72. However, accumulating data show that the contents, size, and membrane composition of EVs are highly heterogeneous and dynamic and depend on the cellular source, state, and environmental conditions 73. Thus, exosome biogenesis plays an important role in its ability to transfer contents to recipient cells. At the same time, EVs enter recipient cells via a variety of mechanisms, which adds to their diversity. According to the biological context, the relative importance of the various uptake pathways differs greatly 74, which indicates that the key protein needed for transfer of contents is different for EVs originating from different cell types. Numerous studies have shown that endocytosis is the primary method for EVs uptake. Endocytosis pathways involved include clathrin- and caveolin-dependent endocytosis, clathrin-dependent endocytosis, micropinocytosis, phagocytosis and micropinocytosis, and lipid raft-mediated endocytosis 75. In addition, the surface proteins on EVs can bind and activate receptors on recipient cells. For example, EVs-associated interferon gamma receptor 1 (IFNGR-1) binds free interferon (IFN)-γ via the Stat1 pathway in recipient cells to activate signal transduction 76. Other reports have also shown that EVs can directly fuse with the recipient cell membrane to deliver their cargoes into cells 77.

## The Roles of Extracellular Vesicles in Heart Diseases

### Extracellular Vesicles are Involved in Physiological and Pathological processes of Heart Diseases

#### Extracellular Vesicles in Atherosclerosis

Atherosclerosis, a chronic inflammatory disease of blood vessels, involves multiple processes such as lipid penetration, endothelial dysfunction, inflammatory response, and cell proliferation. Hutcheson *et al.* 78 reported for the first time that EVs mediate the occurrence and development of microcalcification in atherosclerotic plaques. EVs can increase the expression of adhesion molecule receptors in monocytes, which is conducive to the adhesion of monocytes to endothelial cells. At the same time, accumulation of EVs can aggravate the formation of calcifications and promote vasoactive responses. Development of atherosclerosis is initiated by endothelial dysfunction, which is mainly due to local disturbances in blood flow along endothelial cells. Zhang *et al.* 79 found exosomes-mediated miRNA-155 induces endothelial injury and promotes atherosclerosis. Platelet-derived EVs mediate the atherosclerotic interaction of platelets with endothelial cells and monocytes. A recent study showed that activated platelet-derived exosomes can rapidly decrease the expression of type II scavenger receptor CD36 in platelets by enhancing CD36 ubiquitination and proteasome degradation, thereby reducing platelet aggregation and collagen adhesion in the body 80.

#### Extracellular Vesicles in Myocardial Infarction

Rupture of atherosclerotic plaques and subsequent hemorrhage lead to acute myocardial infarction (AMI) 81. During AMI, cardiomyocytes increase secretion of EVs containing heart-specific non-coding RNA, which have a significant protective effect on the heart. For example, miRNA-133 has anti-fibrosis effects, miR-1 has specific antioxidant effects, and miRNA-499 has anti-apoptotic properties 82, 83. Among them, hypoxia-treated cardiac progenitor cell (CPC)-derived EVs can promote angiogenesis after AMI 84.

#### Extracellular Vesicles in Heart Failure

**Figure 2:**
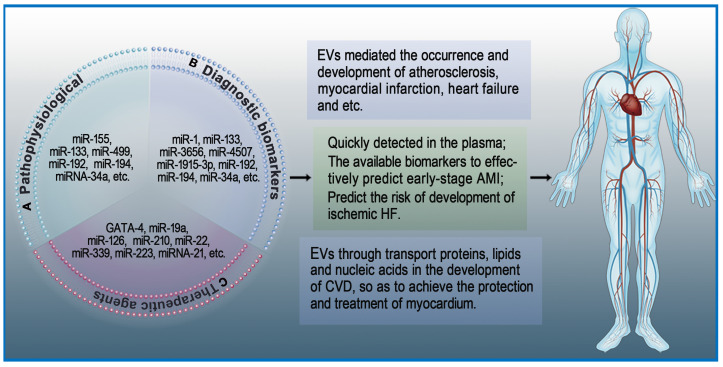
** Roles of extracellular vesicles in heart diseases.** EVs play an important role in the maintenance of cardiac homeostasis and the pathogenesis of heart diseases. A: The role of EVs in pathophysiological processes. B: EVs as diagnostic biomarkers in heart diseases. C: EVs as therapeutic agents in heart diseases.

Continuous damage of numerous cardiomyocytes is an important reason why MI develops into heart failure and eventually leads to death 85. Many studies have shown that EVs secreted from cardiomyocytes are involved in the process of heart failure 86. Matsumoto *et al.* 87 found that circulating exosomal miRNAs (miRNA-192, miRNA-194 and miRNA-34a) are significantly correlated with heart failure after AMI. In another study, Liu *et al.* 88 showed that overexpression of miRNA-132 can protect against apoptosis and oxidative stress in heart failure **(Figure 2A)**.

### Extracellular Vesicles as Diagnostic Biomarkers in Heart Diseases

Cardiac troponins and creatine kinase-MB are classical biomarkers in the diagnosis of AMI. Among these, the 'gold standard' for AMI diagnosis is commonly believed to be cardiac troponins 89. However, this has not stopped the exploration of new biomarkers with higher sensitivity and specificity for diagnosis of early AMI 90. EVs isolated from cardiac cells are partially internalized by neighboring cells, while most of the remaining EVs are released into body fluids. Studies on heart diseases have revealed that EVs isolated from the serum of patients with AMI contain specific mRNA and miRNA 91. For example, the serum levels of miRNA-1 and miRNA-133 in patients with acute coronary syndrome (ACS) are elevated. These elevated miRNA-1 and miRNA-133 have been proven to be derived from damaged myocardium, and they are likely to be stored in EVs 92. Deddens *et al*. 93 demonstrated that heart- and muscle-specific miRNAs are transported by EVs and can be quickly detected in the plasma. Since these EVs are rich in released miRNAs and their detection precedes the expression of traditional damage markers, they have a strong possibility to become early biomarkers of AMI. Su *et al*. suggested that serum exosomal miRNAs (has-miRNA-3656, has-miRNA-4507, and has-miRNA-1915-3p) can be used for the prediction of AMI at an early stage 94. Additionally, Matsumoto and colleagues found that circulating exosomal miRNAs (has-miRNA-192, has-miRNA-194, and has-miRNA-34a) can predict the risk of developing ischemic heart failure after AMI 95. Similarly, EVs were found to mediate the occurrence and development of microcalcification in atherosclerotic plaques 78. In a study of 488 consecutive patients with various coronary heart disease (CHD) risks, Nozaki *et al*. 96 suggested that endothelial exosomal CD144+ could be an independent predictor of future cardiovascular events and contribute to risk stratification of CHD. Altogether, these studies indicate that exosomal miRNAs can become potential biomarkers of heart diseases** (Figure 2B)**.

### Extracellular Vesicles as Therapeutic Agents in Heart Diseases

The adult mammalian heart is a terminally differentiated organ, meaning cardiomyocyte injury is difficult to repair 97. As an important transmission system in vivo, EVs regulate gene expression in target cells through transport proteins, lipids, and nucleic acids during the development of heart diseases, thereby protecting and treating myocardium 98. Early research showed that overexpression of GATA-4 increases mesenchymal stem cells (MSCs) differentiation into cardiac cell phenotypes as well as promotes the survival of MSCs in ischemic environments 99. Yu *et al*. 72 suggested that EVs secreted from GATA-4-overexpressing MSCs could deliver miRNA-19a into myocardium and produce a greater cardioprotective effect. Hypoxia-inducible factor 1 (HIF-1) is an oxygen-sensitive transcription factor that has great significance in angiogenesis 100. EVs secreted from HIF-1-overexpressing CPCs have been shown to deliver miRNA-126/miRNA-210 and increase angiogenic responses in the hypoxic environment 101. EVs derived from stem cells encapsulate various molecules such as mRNA, miRNA, and proteins that have cardioprotective effects similar to stem cell transplantation. For example, EVs derived from MSCs were found to be enriched with miRNA-22, which directed targeting to methyl CpG binding protein 2 (MECP2) and reduced apoptosis of cardiomyocytes due to ischemia 102.

Cardioprotective effects can also be derived from endogenous EVs in vivo. During oxidative stress, cardiomyocytes increase the synthesis and secretion of EVs. Garcia *et al*. suggested that EVs secreted by cardiomyocytes can stimulate endothelial cells to produce vessels under glucose-deprived culture conditions 33. Platelet-derived EVs can also affect endothelial cells and platelet function in CVD. Tan *et al.* 103 showed that active platelet-derived EVs delivering miRNA-339, miRNA-223, and miRNA-21 inhibit the expression of platelet-derived growth factor receptor-beta (PDGFRβ) in vascular smooth muscle cells (SMCs) and increase the number of capillaries in ischemic myocardium. Li and colleagues 104 indicated that anti-IL-1 platelet-derived EVs remove cytotoxic IL-1 and repair ischemic myocardium during AMI. Li *et al.* suggested that coronary serum exosomes in patients with MI regulate angiogenesis through miR-939-mediated nitric oxide signaling pathway 105. Another interesting new research direction is cardio-renal exosome-derived miRNA-1956, which regulates the activation of paracrine VEGF signaling in adipose-derived MSCs after AMI 106 **(Figure 2C)**.

## Challenges in the Delivery of Extracellular Vesicles to the Heart

Due to their unique advantages, EVs can be positioned as efficient drug carriers 107. The special structure of EVs can protect their contents from degradation in the extracellular environment for a long time 73, and their surfaces contain special lipids and proteins that are conducive to fusion with recipient cells and subsequent release of cargo including drugs 41, 108, 109. EVs containing short hairpin RNA plasmids and interfering RNA inserted by conventional mass electroporation have shown higher therapeutic efficacy in inhibiting targets than synthetic nanocarriers in conventional preclinical studies. However, insertion of large amounts of RNA into EVs is still technically challenging and may be limited to specific cell types. Recently, Yang *et al*. 110 discovered a type of cellular nanoperforation (CNP) that can effectively integrate high levels of mRNA into EVs for targeted transcriptional operations and therapy. Compared to mass electroporation and other EV production methods, CNP can produce up to 50-fold more EVs from cells with low basal secretion levels, and mRNA transcripts can be increased by more than 103-fold. However, Kooijmans *et al.* 111 stated that electroporation disrupts EVs integrity and siRNA loading is accompanied by substantial siRNA aggregate formation, which may lead to overestimation of the amount of siRNA actually loaded into EVs. Similar to EVs, liposomes are synthetic vesicles with phospholipid bilayer structures that can be loaded with a variety of proteins, nucleic acids, and drug molecules. A comparison of liposomes and EVs as drug delivery vehicles is provided in **Table 1**. Current studies have shown that EVs provide potentially superior drug delivery than liposomes 112.

The expected biological effects of EVs are mostly produced from internalization by recipient cells through endocytosis pathways 113. Studies have shown that EVs administered intraperitoneally, subcutaneously, and intravenously are quickly cleared from the blood circulation and subsequently accumulate in the lung, spleen, liver, and gastrointestinal tract 114, 115 **(Figure 4)**. Regardless of the delivery route and cell source, most systemically injected EVs are rapidly absorbed by macrophages in the reticuloendothelial system and excreted from the body 116-118. In a previous study, to counteract non-specific delivery, the authors used approximately ten times the normal dose used for intramyocardial injections 119. Another study used intracoronary and intramyocardial injections and showed that intramyocardial delivery was more effective 120. Although intramuscular injections can be performed in animal studies, such a situation is more complicated in a clinical setting and requires a physician to perform the catheterization 121.

**Figure 3:**
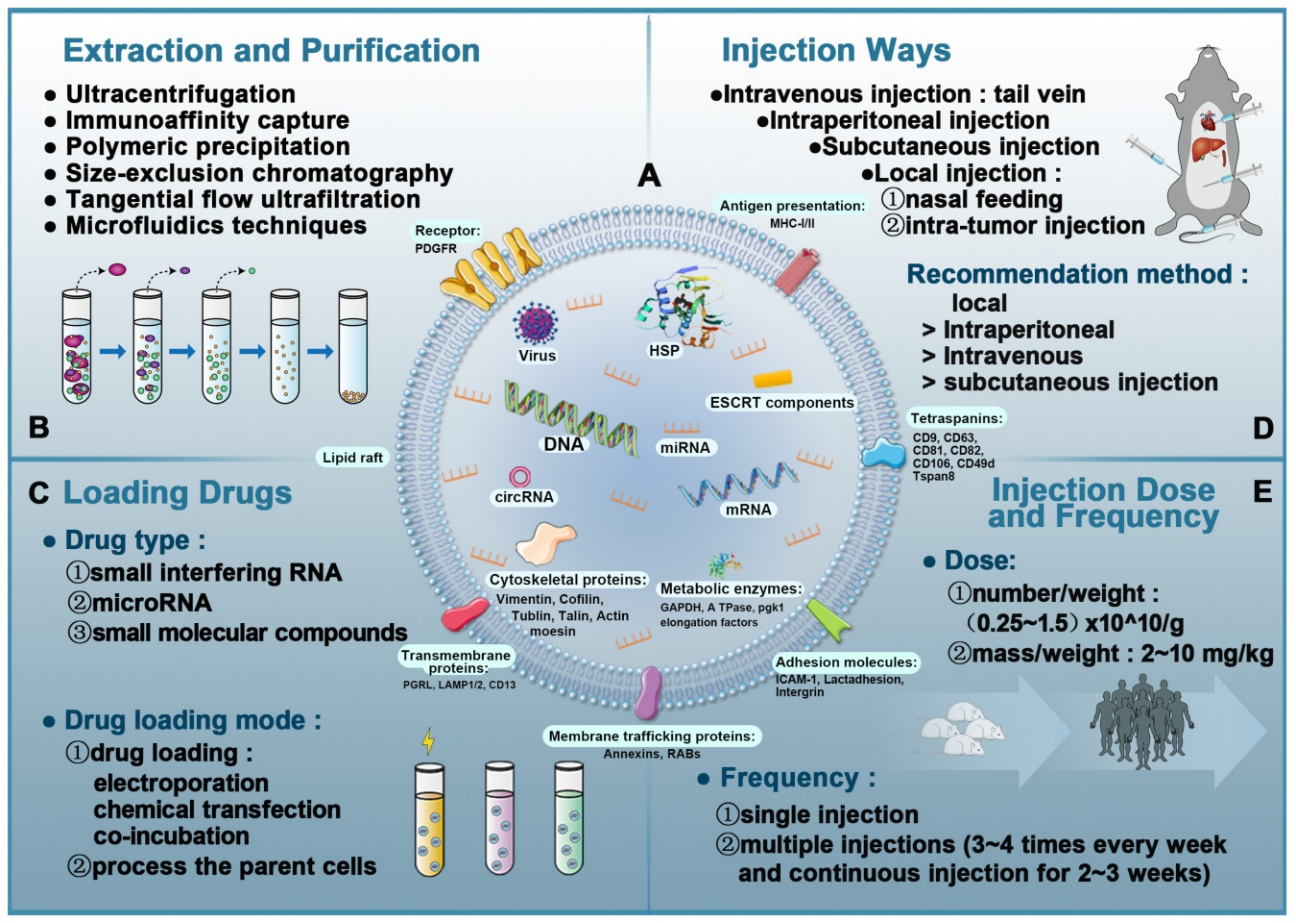
** Overview of extracellular vesicles, their composition, isolation, and analysis in vivo.** A: The composition of exosomes (including proteins, lipids, and nucleic acids). B: Exosomal isolation and purification techniques. C: Drug-loading techniques to produce EVs-based nanotherapeutics. D: Administration routes in EVs-based nanotherapeutics. E: Injection dose and frequency in EVs-based nanotherapeutics.

**Figure 4:**
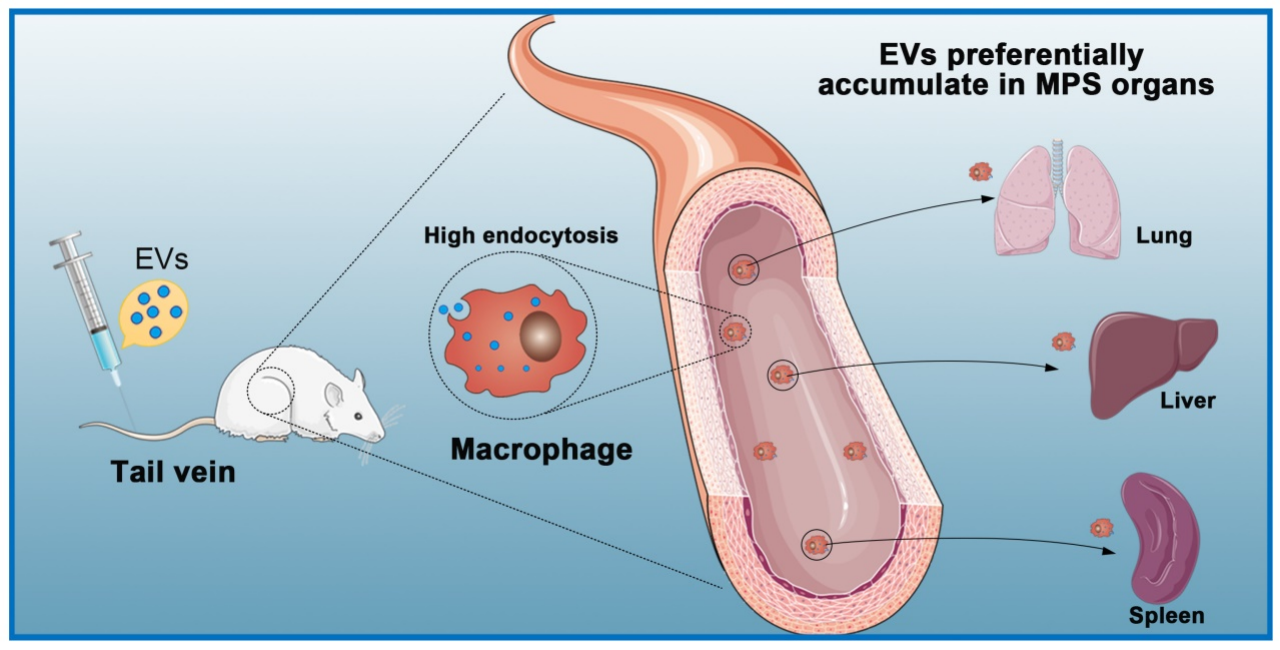
** Macrophages eliminate circulating extracellular vesicles.** Injected EVs are quickly cleared by macrophages of the mononuclear phagocyte system (MPS) and preferentially accumulate in MPS organs (e.g., liver, spleen, lung).

EVs extraction and purification do not have universally recommended techniques. Currently, there are six major methods: ultracentrifugation, immunoaffinity capture, polymeric precipitation, tangential flow ultrafiltration (TFU), microfluidics techniques, and size-exclusion chromatography (SEC) **(Figure 3B)**. Based on investigations by the International Society for Extracellular Vesicles (ISEV) in 2015, ultracentrifugation is the most widely adopted and reliable method and considered to be the gold standard for EVs extraction. Each technique has a unique set of advantages and disadvantages, as shown in **Table 2**. Another pertinent question is the cost of extracting EVs. EVs are secreted by cells, so their production depends on the ability to produce large numbers of cells without changing their phenotype 122. Moreover, it is not only difficult to produce large quantities of EVs, it is also challenging to produce EVs with high purity and stable quality.

The typical yield of EVs isolated from 1 mL of culture medium could be less than 1 µg of EV protein. As such, therapeutic doses of EVs (~10-100 µg of protein) can be achieved in mouse models 123, 124. For humans, the effective dose is an order of magnitude more than the dose used in mouse models to compensate for the rapid elimination of EVs from the body. Owing to the high incidence rate of heart diseases, there is an urgent need to improve the specificity of EVs delivery to cardiomyocytes to reduce consumption by non-specific delivery 125. **Figure 3C-E** shows the details of EV-based nanotherapeutics including drug loading techniques, administration routes, and injection dose and frequency.

## Targeted Delivery of Therapeutic Extracellular Vesicles in Heart Injury

### Local Delivery of Hydrogels Encapsulating Extracellular Vesicles

Hydrogels have been widely used to create drug delivery systems with ideal therapeutic effects 126. Hydrogels are suitable for biological applications due to their large water content and biocompatibility. They typically have excellent malleability and are similar to the natural extracellular matrix (ECM). Moreover, the physical properties of hydrogels can be controlled to adjust the rate of matrix degradation to release encapsulated cargo. In the past few decades, a lot of research has focused on hydrogels, and significant progress has been made in their design, synthesis, and use in many biological and biomedical applications 127 including delivery of EVs 128. Hydrogels can provide practical options for delivering large numbers of EVs to target sites. Qin et al. 129 first described the concept of encapsulating EVs in hydrogels and confirmed that the delivery system can significantly enhance bone formation in vivo.

In heart diseases, the ability to release EVs over a long duration may be more practical than repeated implantation of fresh hydrogels into the heart with release of EVs over a short duration. **Table 3** summarizes studies on the duration of EV release from hydrogels in the heart. Chen *et al*. 130 found that endothelial progenitor cells (EPCs)-derived EVs encapsulated in shear-thinning hydrogel (STG) could be injected into the ischemic myocardium, where they promoted angiogenesis and improved myocardial hemodynamics in a rat MI model. Additionally, STG improved therapeutic efficiency and the efficacy of EVs-mediated myocardial protection. Hydrogel encapsulation localized the EVs to the myocardial ischemic border area, where they could be observed for 21 days. In a separate study by Han *et al*. 131, the authors loaded EVs secreted by human umbilical cord MSCs (HUC-MSCs) into a peptide-based hydrogel called PGN. The authors showed that the PGN hydrogel ensured stable and sustained release of EVs at the myocardial ischemic border area over 21 days. Additionally, the EV-PGN hydrogel treatment better improved cardiac function than EVs alone and also reduced fibrosis, apoptosis, and inflammation. Laponite^®^ is a smectite nanoclay composed of discoidal nanoparticles that can solve the biocompatibility problems associated with carbon-based nanoparticles. Owing to its high surface area-to-volume ratio and disk-shaped charged surface, Laponite^®^ possess good loading capacity for growth factors 132, 133. Waters *et al*. 134 created a hydrogel of Laponite® and gelatin (nSi) as a vehicle for the delivery of EVs. The authors showed that the nSi hydrogel can sustain high strain and reduced EVs effusion out of the therapeutic site for 21 days after injection. In another study, Liu *et al.* 135 engineered a hydrogel patch to slowly release iPSC-derived cardiomyocyte EVs, which have been shown to promote recovery within 24 h after MI and reduce arrhythmia in humans. The hydrogel-delivered EVs recovered the normal physiological activity of cardiomyocytes and reduced the infarct size 4 weeks after MI implantation in diseased rats. Furthermore, Huang *et al.* 136 developed an off-the-shelf therapeutic cardiac patch composed of a decellularized porcine myocardial extracellular matrix scaffold and synthetic cardiac stromal cells (synCSCs) generated by encapsulating secreted factors from isolated human cardiac stromal cells. The transplanted artCP promoted cardiac recovery by reducing scarring, promoting angiogenesis, and boosting cardiac function in both rat and porcine models of AMI. Interestingly, these cases indicate that some hydrogels alone have therapeutic effects. For example, the PGN hydrogel described above is a functional peptide hydrogel based on a growth hormone-releasing peptide (His-DTrp-Ala-Trp-DPhe-Lys-NH2) that activates pro-survival pathways and inhibits inflammation and fibrosis 131. In addition, Chen *et al.* 130 found that STG treatment alone significantly improved the end-systolic pressure volume relationship 4 weeks after MI compared with PBS control. This effect was due to the hyaluronic acid (HA) composition of STG, as HA is a biologically active pro-angiogenic molecule that can affect the proliferation, migration, and tubule formation of endothelial cells through CD44- and HA-mediated cell movement signal receptors.

In short, biodegradable and highly porous hydrogels can provide continuous treatment of heart tissue with a matrix of vesicles. By placing hydrogels loaded with EVs directly at or around the target site, hydrogels also prevent the disappearance of EVs from the target site. With hydrogels, only a small number of EVs are required to achieve therapeutic effects. In comparison, a large number of EVs must be injected intravenously to counter the poor systemic bioavailability of EVs 137-144.

### Genetic Engineering of Extracellular Vesicles for Therapeutic Delivery

Compared with other gene delivery vectors, EVs are non-mutagenic, less immunogenic, and non-cytotoxic. These characteristics indicate that EVs can become an ideal therapeutic carrier 145. In addition, genetic engineering can modify EVs to improve their therapeutic efficiency and targeting ability by displaying homing peptides or ligands on their surface. Although genetic engineering of EVs does not change their biological distribution time, it does shorten the time required by EVs to reach their therapeutic target and significantly reduces off-target effects, thereby improving the therapeutic effect 146. Transmembrane proteins have been shown to accumulate in EV compartments, and targeting EVs to specific sites is achieved by displaying ligands/homologous peptides that can be fused to EV surface proteins 147. There are ample opportunities to explore the potential uses of EVs in targeted therapies because phage display and in vivo biological screening techniques can target specific sites on many peptides 148. Zhu *et al*. 8 modified EVs secreted from hypoxia-conditioned MSCs with an ischemic myocardium-targeting peptide (CSTSMLKAC), thereby preferentially targeting ischemic injured cardiomyocytes and minimizing off-target effects. In another study, Vandergriff *et al*. 149 showed that modification with CSTSMLKAC, here called cardiac homing peptide (CHP), improved the efficacy and reduced the effective dose of EVs delivered intravenously. To generate an effective EVs delivery strategy that can target cardiomyocytes, Mentkowski *et al*. 150 designed cardiomyocyte-derived cells (CDC) that express LAMP2B (an EV-addressed membrane protein) fused with a cardiomyocyte-specific peptide (CMP; WLSEAGPVVTVRALRGTGSW). EVs isolated from the engineered CDCs expressed CMP on their surface and maintained their true physical properties. Compared to non-targeted EVs, the targeted EVs reduced cardiomyocyte apoptosis after cardiomyocyte injection, increased cardiomyocyte uptake, and improved cardiac retention. In another study, Zhang *et al*. 151 enhanced the efficiency of EVs transport in ischemia-injured myocardium by engineering EVs with monocyte mimics (monocyte/macrophage membrane vesicles) using membrane fusion. An intriguing new study 152 suggests that platelet-derived EVs fused with cardiac stem cells (CSCs) can selectively bind collagen-coated surfaces and endothelium-denuded aortas. Therefore, these CSCs have natural targeting and repair capabilities to the damaged area of MI. In addition, Yim *et al*. 153 developed a strategy called “exosomes for protein loading via optically reversible protein-protein interactions (EXPLORs)” for intracellular delivery of target proteins. By integrating a reversible protein-protein interaction module controlled by blue light with the endogenous process of exosome biogenesis, the authors were able to successfully load cargo proteins into newly generated exosomes, which offered efficient intracellular delivery of soluble proteins into recipient cells. The authors demonstrated transfer of mCherry, Bax, super-repressor IκB protein, and Cre enzyme as functional proteins into target cells in vitro and into brain parenchymal cells in vivo. Increasing data show that soluble proteins play critical roles in the therapeutic effects of EVs.

Altogether, these EV engineering strategies might offer better ways to assess the effects of EVs and provide novel technologies to help clinicians better manage regenerative therapeutics for heart diseases.

### Two-step Extracellular Vesicles Delivery Strategy

Even though EVs have various advantages compared to existing delivery systems, such as lower immunogenicity and higher affinity, their distribution to the heart is limited by rapid clearance from the blood by the MPS and subsequent accumulation in the liver and spleen 154. The plasma half-life of EVs is only 70-80 min 155, 156. In one study, fluorescently labeled EVS injected into the tail vein of mice were mainly captured by the liver, spleen, and lung, as well as kidney, bone marrow, and other organs. Additionally, 4 h after intravenous injection of EVs in mice, 28%, 7%, and 1.6% of fluorescence activity was detected in the liver, lung, and spleen, respectively 155. However, EVs were mainly absorbed by macrophages in the liver and spleen. There are multiple processes by which macrophages take up EVs, such as micropinocytosis, endocytosis, phagocytosis, and plasma membrane fusion 157. Whether endocytosis or phagocytosis is the dominant process in EVs uptake by macrophages remains controversial. Clathrin has great significance in the formation of vesicles. Clathrin is a hexamer of proteins, three light and three heavy chains, that can assemble into a basket-like lattice spontaneously to promote the budding process of endocytosis 158. The CLTC gene encodes clathrin heavy chain 1 159. Wan and colleagues were the first to show that CLTC plays a significant role in EVs uptake by the MPS. Inhibition of CLTC with siRNA was shown to significantly block endocytosis mediated by the MPS in the spleen and liver, thereby increasing the delivery of intravenously injected EVs in the heart 117. In the therapeutic anticancer area, Belhadj* et al*. 160 exploited a combined “eat me/don't eat me” tactic to reduce endocytosis of macrophages. For the “eat me” component, EVs extracted from DC2.4 cells were modified with cationic mannan to saturate the MPS. For the “don't eat me” component, CD47-enriched exosomes from human serum were fused to nanocarriers to avoid MPS phagocytosis. This combined tactic reduced endocytosis of EVs by macrophages, extended their circulation time, and increased tumor accumulation of EVs by 123.53% in comparison with conventional nanocarriers.

## Conclusions and Perspective

**Figure 5:**
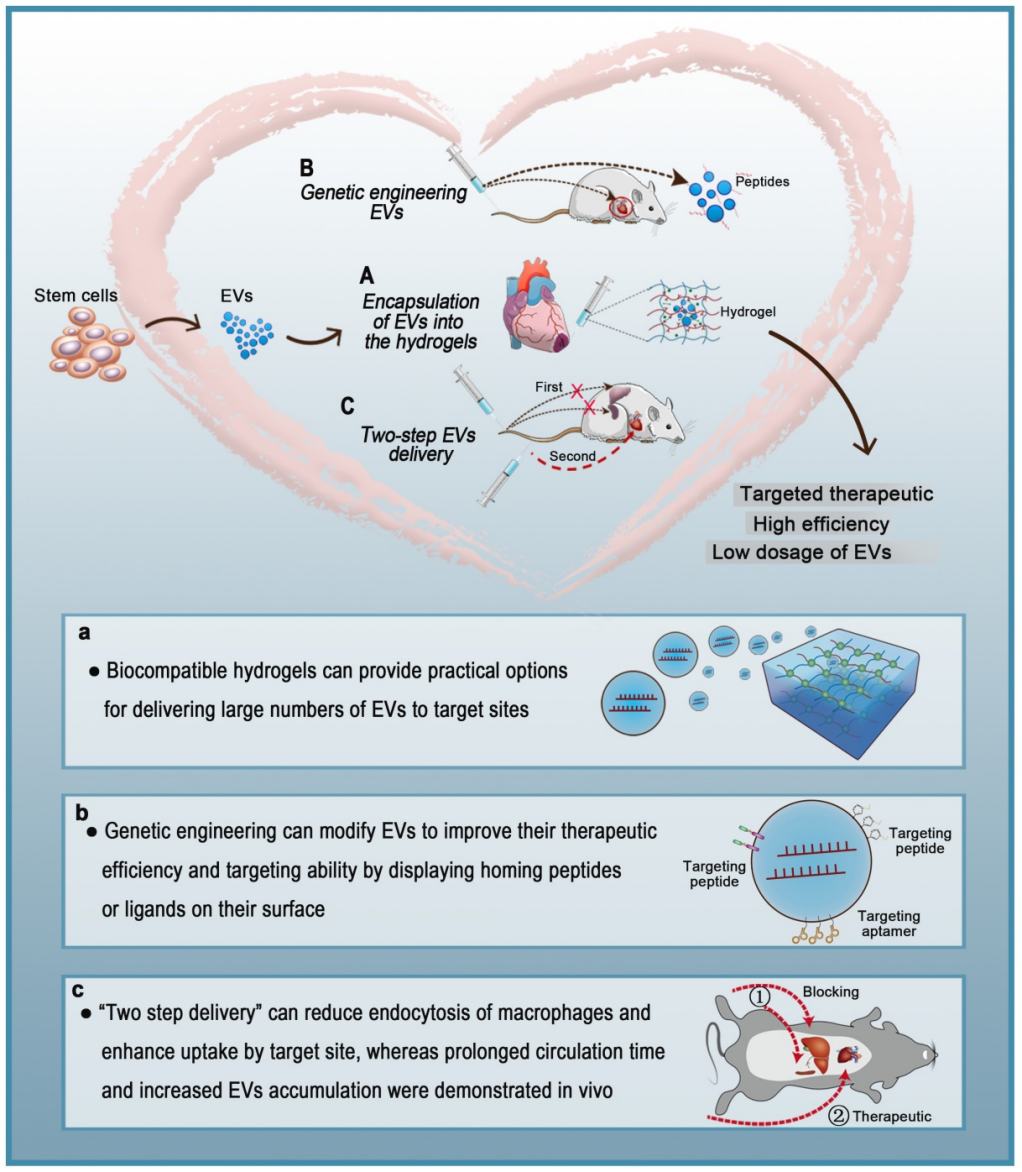
** Targeted therapeutic delivery of extracellular vesicles in heart diseases.** Three strategies for targeted delivery of therapeutic EVs to the heart. A: Encapsulation of EVs in hydrogels. B: Genetic engineering of EVs. C: Two-step EVs delivery.

Various types of cells secrete EVs, which can act as vehicles for regulating intercellular communication and gene delivery 161, 162. As naturally derived nanovesicles, EVs have increased stability and biocompatibility, as well as lower toxicity and immunogenicity than synthetic nanocarriers 17. However, multiple studies have shown that EVs injected intravenously are rapidly removed by macrophages of the MPS and accumulate in organs such as the liver, spleen, and lungs 154. Such challenges in the efficient delivery of EVs to target sites are yet to be solved. To date, three strategies for the targeted delivery of therapeutic EVs to the heart have been reported: 1) encapsulation of EVs in hydrogels, 2) genetic engineering of EVs, and 3) two-step EVs delivery **(Figure 5)**. Each strategy has a unique set of advantages and disadvantages **(Table 4)**. Hydrogels have been widely used in biomedical research for drug delivery to tissues, as well as cell-based therapies and tissue engineering. Hydrogels have emerged as attractive biomaterials due to their excellent biodegradability and biocompatibility. The most important advantage of hydrogels is their ability to provide an excellent condition for maintaining the integrity of EVs. Recently, various forms of hydrogels such as chitosan hydrogel, imine cross-linked hydrogel, and chitosan/silk hydrogel have been used to improve the therapeutic efficacy of EVs in different medical fields 143, 163, 164. However, this strategy also has challenges; for example, it is difficult to identify cross-linking agents that do not participate in intracellular chemical reactions and at the same time produce effective hydrogels. Alternatively, surface modification methods (aka “EV engineering”) can be used to enhance the specific binding of EVs to receptors. This method can be used to link heart-targeting peptides to EVs to help reduce the effective dose for intravenous administration. However, since the liver and spleen are the main receptors and target organs of EVs, they are very vulnerable to adverse effects. Therefore, it is necessary to avoid non-specific accumulation of EVs in the spleen and liver so as to improve the delivery efficiency of EVs to the target sites. The two-step EVs delivery strategy is a promising gene therapy method. Macrophage saturation with EVs in advance can successfully block subsequent endocytosis of therapeutic EVs and effectively improve their distribution in the heart. However, the effects of this method are not yet clear and further theoretical and experimental studies are required. Although EV-based theragnostic treatments have made revolutionary progress over the past few decades, there are still unresolved challenges in the field. In summary, strategies for the targeted delivery of EVs to the heart will effectively shorten the arrival time of EVs to the target site, prolong the survival time of EVs, and improve their therapeutic effects.

## Figures and Tables

**Table 1:** Advantages and disadvantages of liposomes and EVs.

	Liposomes	EVs
Origin	Artificially produced from a wide variety of phospholipids	Secreted by cells
Shape and size	Phospholipid bilayer structures; ~100 nm and homogenous	Phospholipid bilayer structures including many proteins (e.g., tetraspanins); 30-120 nm
Manufacture	Many manufacturing possibilities	No current manufacturing methods
Contents	Single component	Complex components (e.g., DNA, RNA, proteins, small molecule drugs)
Drug loading capacity	High loading of hydrophilic drugs	Low drug loading
Half-life	10-55 h	70-80 min
Targeted delivery	Poor and dependent on the enhanced permeability and retention (EPR) effect	Strong with ability to cross biological membranes and high endogenous targeting potential

**Table 2:** Comparison of EVs extraction and purification techniques.

Strategy	Principle	Advantages	Disadvantages
Ultracentrifugation	Density, size, and shape	Low cost Low risk of pollution Fast Scalable	Low purity Damages membrane integrity Time consuming Labor intensive
Immunoaffinity capture	Specific recognition of exosome markers by corresponding immobilized antibodies	Suitable for separating exosomes of specific origin High purity	High cost Low yield Damages membrane integrity
Polymeric precipitation	High hydrophilic water-excluding polymers	Low cost Scalable Simple	Low purity Protein aggregates remain
Tangential flow ultrafiltration	Size and shape	High purity Fast Scalable Simple	Low purity Membrane-fouling High cost
Microfluidics techniques	Immunoaffinity, size, and density	Low cost Fast Simple Easily automated and integrated with diagnosis	Low sensitivity and specificity Small sample size
Size-exclusion chromatography	Size	High purity Fast Maintains membrane integrity	High cost Needs specialized equipment and filler

**Table 3:** Hydrogels used to encapsulate EVs for treatment of myocardial infarction.

Type of Hydrogel	Materials	Cell-derived EVs	Function	EVs Preservation in Heart	Advantages	Disadvantages	Ref
Shear-thinning (STG)	adamantane- and b-cyclodextrin-modified hyaluronic acid	Endothelial progenitor cells	Improve angiogenesis and promote function	21 days; Slow release	Prolonged therapeutic duration, slow elution of EVs, and high local concentrations; Translation to the clinical setting	Increased inflammation in the ischemic border	Chen et al. (2018)
PA-GHRPS (PGN)	Peptide amphiphile, cardiac protective peptides, matrix metalloprotease-2	Human umbilical cord mesenchymal stem cells	Promote cardiac repair	21 days; Enhanced retention and stability	Non-immunogenic and relatively small pore size; Easily tunable	Difficult to control release	Han et al. (2019)
Nanocomposite (nSi)	gelatin, Laponite	Human adipose-derived stem cells	Repair injured cardiac tissue	21 days; Controlled release of growth factors present in EVs	High surface-to-volume ratio and discoidal charged surface; Biocompatible	Biodegradation in myocardial tissue is unknown	Waters et al. (2017)
Hydrogel patch	Rat tail collagen type I, Gelfoam mesh	Induced pluripotent stem cells	Promote recovery of the heart	21 days; Sustained release	Well-defined neutral material	Larger pore size; More invasive injury	Liu et al. (2018)
							

**Table 4:** Current strategies for targeted delivery of therapeutic EVs to the heart.

Strategy	Principles	Advantages	Disadvantages
Encapsulation of EVs in hydrogels	Injectable hydrogel to localize EVs in the myocardial ischemic border	Low dosage of EVs Low cost Avoids repeated implantation Efficient heart-targeted delivery of EVs Stable and sustained release of EVs Hydrogels have a therapeutic effect on cardiovascular diseases Easy to use	Epicardial injection Risk of angiemphraxis Potential toxicity of residual unreacted cross-linkers Uncertain release profiles in vivo
Genetic engineering of EVs	Surface modification of EVs with homing peptides or ligands that target the heart	Low dosage of EVs Avoids repeated implantation Efficient heart-targeted delivery of EVs Intravenous administration	High-tech equipment required Labor intensive High cost Unstable release of EVs Low efficiency EVs with a shorter duration
Two-step EVs delivery	Blockage of MPS uptake followed by delivery of therapeutic EVs	Avoids repeated implantation Localizes EVs to the target site Intravenous administration Efficient heart-targeted delivery of EVs Blocks the endocytic function of the MPS in the spleen and liver	Time consuming Labor intensive Unstable release of EVs Uncertain efficiency EVs with a shorter duration

## Abbreviations

EVs: extracellular vesicles
AMI: acute myocardial infarction
CHD: coronary heart disease
HIF-1: hypoxia-inducible factor 1
CDC: cardiomyocyte-derived cells
CHP: cardiac homing peptide
CMP: cardiomyocyte-specific peptide
CNP: cellular nanoperforation
DUBs: deubiquitinating enzymes
ECM: extracellular matrix
EPCs: endothelial progenitor cells
ESCRT: endosomal sorting complexes required for transport
ESEs: early-sorting endosomes
iCMs: induced cardiomyocytes
ILV: intra-luminal vesicles
MPS: mononuclear phagocyte system
MVB: multivesicular bodies
NEM: *N*-ethylmaleimide
STG: shear-thinning hydrogel
